# Development of nomogram model based on LASSO-Logistic regression for predicting postoperative undernutrition to complex anorectal malformation: a pilot exploratory study

**DOI:** 10.1186/s12876-025-04202-5

**Published:** 2025-08-16

**Authors:** Wei Feng, Linxiao Fan, Jinping Hou, Xiaohong Die, Yi Wang, Rui Jiang

**Affiliations:** 1 Department of General & Neonatal Surgery, Children’s Hospital of Chongqing Medical University, National Clinical Research Center for Child Health and Disorders, Ministry of Education Key Laboratory of Child Development and Disorders, Chongqing Key Laboratory of Structural Birth Defect and Reconstruction, Chongqing, China; 2 Department of Anesthesiology, Children’s Hospital of Chongqing Medical University, National Clinical Research Center for Child Health and Disorders, Ministry of Education Key Laboratory of Child Development and Disorders, Chongqing Key Laboratory of Structural Birth Defect and Reconstruction, Chongqing, China

**Keywords:** Complex anorectal malformation, Undernutrition, Nomogram model, Lasso-Logistic regression

## Abstract

**Background:**

Nutritional problems in patients with anorectal malformation (ARM) after anorectoplasty have not received sufficient attention. This study aimed to establish prediction model of postoperative undernutrition for patients with complex ARM.

**Methods:**

Retrospective review of 104 patients with complex ARM was conducted with assessments of clinical data. Lasso-Logistic regression analysis was used to identify independent factors, and then establish a Nomogram model for predicting postoperative undernutrition. Harrell’s concordance index and calibration curves were applied to evaluate the performance of this model. R software was used for statistical analysis.

**Results:**

Of the 104 patients, the proportion of malnutrition was 28.85% (30/104). Lasso-Logistic regression analysis showed that non-parental caregivers (Odds ratio [OR]: 11.20), undernutrition at anorectoplasty (OR: 4.101), surgery for other systemic malformation (OR: 8.378), and feeding method (artificial, OR: 25.320) were independently associated with postoperative undernutrition (all *P* < 0.05), and Nomogram model was developed based on these determinants. The area under the receiver operator characteristic curve of this cohort was 0.877 and it still was 0.906 through bootstrapping validation (*n* = 1000). The H-L goodness-of-fit test showed that there was no significant difference between the predicted and actual incidence of postoperative undernutrition (χ2 = 7.55, *P* = 0.273). Meanwhile, the calibration curves indicated that the forecast was in good agreement with the actual situation. Decision curve showed that when the probability threshold of Nomogram model predicting malnutrition was >0.03, application of the model would add net benefit compared to either the treat-all strategy or the treat-none strategy.

**Conclusion:**

This Nomogram model for predicting postoperative undernutrition of complex ARM patients showed desirable accuracy and discrimination for clinicians, aiding the early initiation of preventative interventions for undernutrition.

**Supplementary Information:**

The online version contains supplementary material available at 10.1186/s12876-025-04202-5.

## Introduction

Congenital anorectal malformation (ARM) is a congenital condition involving the anus and rectum, with a prevalence of approximately 3.26 per 10,000 births in the population [1]. Complex ARM, including imperforate anus without fistula, rectobulbar fistula (RB), rectoprostatic fistula (RP), rectobladderneck fistula, and cloacal malformations, are challenging for pediatric surgeons in preoperative diagnosis, surgical techniques, and postoperative management [2]. Over the past several decades, the development of nursing care and surgical techniques have significantly reduced the mortality of complex ARM, but a series of problems after anorectoplasty have gradually attracted attention, among which the most prominent concerns are defecation function and quality of life [3, 4]. However, little exists in the current literature on postoperative nutritional status assessment in patients with complex ARM.

As a tertiary referral center, we have observed that the incidence of postoperative undernutrition was not optimistic in the standardized and systematic follow-up. Postoperative undernutrition negatively affects the growth and development of the child, even into adulthood [5]. An understanding of the risk for undernutrition in this population will elucidate the determinants of disparities, as well as inform early interventions by pediatric surgeons and caregivers. Therefore, by collecting clinical data of patients with complex ARM, this study expected to assess the incidence of postoperative undernutrition and established clinical prediction model to evaluate the risk of undernutrition.

## Materials and methods

### Study approval

This study was approved by the Institutional Research Ethics Board of Children’s Hospital affiliated Chongqing Medical University (Date: 2023/No: 01) and complies with the 1964 Helsinki Declaration and its later amendments or comparable ethical standards. Because this study was retrospective, the requirement for informed consent was waived.

### Study population

**Fig. 1:**
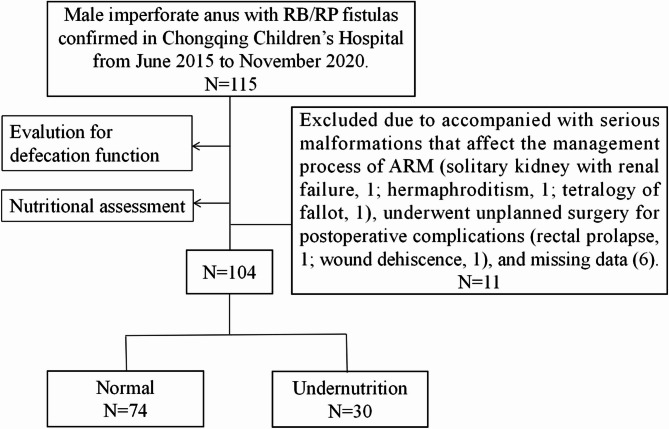
Flow chart of the study population

This is a retrospective and exploratory study. We reviewed the files of patients who had received posterior sagittal anorectoplasty (PSARP) for treating male imperforate anus with RB/RP in General & Neonatal Surgery Department of Children’s Hospital affiliated Chongqing Medical University (a tertiary pediatric hospital and National Clinical Research Center for Child Health and Disorders in China) from June 2015 to November 2020 consecutively. Furthermore, only patients who had complete clinical data and cooperated with the follow-up process were included. Patients were excluded if they received unplanned surgery due to postoperative complications (rectal prolapse, 1 case; wound dehiscence, 1 case); accompanied with serious malformations affecting the standard management process of ARM (solitary kidney with renal failure, 1 case; hermaphroditism, 1 case; tetralogy of fallot, 1 case); and missed follow-up information (6 cases). Figure 1 shows a flow diagram for the inclusion and exclusion of patients in this study.

### Study design

According to relevant literature and clinical practice, variables that may influence postoperative nutritional status were retrospectively collected. Clinical data included the following: (1) demographic information: type of ARM (RB or RP), gestational age, birth weight, feeding method (breast, artificial, or mixed feeding); (2) social factors: residence (urban or rural), relationship of caregiver (parents or others), educational level of caregivers; (3) associated malformations: congenital heart defect (CHD), multiple congenital disorders (MCD), surgery for other systemic malformations; (4) defecation function: all patients were evaluated for defecation function at about 1 year old, that is, about 3 months after colostomy closure. Furthermore, we recorded four-time points: age at the time of colostomy, PSARP, colostomy closure, and last nutritional assessment (> 3 years old). It needs to be stated that all patients in this study underwent three-staged repair, as described detailedly in our previous report [6].

### Definition

Weight and height measurements were recorded for all children according to standard methods. These measurements were compared with the Chinese Child Growth Standards and were expressed as standard deviation scores (Z-scores) to adjust for age and gender simultaneously (including height-for-age [HAZ] and weight-for-age [WAZ]). According to the WHO criteria, categorical outcomes were defined as follows: malnutrition, HAZ and (or) WAZ < −2; at risk of malnutrition, HAZ and (or) WAZ ≥−2 and <−1 [7]. with the aim of facilitating better application in clinical practice, we classified patients at risk of malnutrition and malnutrition as the undernourished group [8, 9]. It should be noted that we focused on the factor of undernutrition, including malnutrition and at risk of malnutrition, so patients who did not match these definitions were classified as “normal”.

Defecation function was evaluated using Rintala’s modified questionnaire, conducted by two experienced surgeons (Yi Wang and Wei Feng). Because we assessed defecation function at 1 year of age, we used a modified Rintala’s questionnaire that did not include the parameter of social problems. Modified Rintala’s questionnaire, for children younger than 3 years, consists of 6 questions: ability to hold back defecation, feels/reports urge to defecate, frequency of defecation, soiling, fecal incontinence, and constipation. Patients were classified according to their scores into four categories, as follows: normal (15 ~ 17), good (9 ~ 14), fair (4 ~ 8), and poor (0 ~ 3) [10].

CHD, diagnosed by echocardiography result, is classified into 3 forms based on the presence and severity: no (no abnormality), minor (incomplete foramen ovale closure with a significant shunt, secundum atrial septal defect, and/or small ventricular septal defect), and major (the remaining defects) [11, 12]. It’s worth noting that patent ductus arteriosus was defined when the condition persisted after 1 month of life or when cardiac surgery was performed before the first month of life. In addition, the atrial septal defect was defined as a heart defect in which blood continues to flow between the atria even after 6 months of age.

MCD was defined as the presence of three or more congenital malformations, including ARM, or if they had a syndrome that was confirmed by a clinical geneticist [1, 13].

### Statistical analysis

R software (version 4.3.2; https://www.r-project.org/) was used for statistical analysis. Categorical data was expressed by n (%), and the chi-squared test was used for comparison. The numerical data were assessed for normality by the Shapiro-Wilk test: if they matched, they were expressed as mean ± standard deviation (SD) with Student’s t-test; if they did not match, they were expressed as quartile spacing with Mann-Whitney test.

First, to enhance the stability of our Nomogram model and avoid over-fitting the results, we employed least absolute shrinkage and selection operator (LASSO, “glmnet” package) regression for variable selection based on the cohort (lambda. min). The postoperative nutritional status was taken as the dependent variable, and the covariates were taken as the independent variables to input the LASSO regression model. In this way, optimal regularization parameter λ was determined by cross-validation to prevent overfitting and solve the problem of collinearity [14]. Then, the variables with corresponding coefficients that were not zero were included in the multivariable logistic regression analysis (“glm” packag) for screening independent factors. Odds ratios (ORs) with 95% confidence intervals (95% CIs) were calculated for significant predictors. Then, the “rms” software package was used to establish Nomogram model for predicting postoperative undernutrition, and the receiver operating characteristic curve (ROC), area under the curve (AUC), decision curve analysis (DCA), and clinical impact curve (CIC) analysis were plotted to evaluate the discriminatory degree of this model. Furthermore, polynomial equations were extracted using the R package of nomogramEx. The optimal cutoff function and the Youden index were used to determine the thresholds for the two risk stratifications of the model. To quantify the discrimination performance of the model, Harrell’s concordance index was measured (bootstrapping validation: 1000 bootstrap resamples). The H-L goodness-of-fit test and the calibration curve were used to evaluate the calibration of this model. Usually, model with an AUC between 0.80 and 0.90 indicated great predictive accuracy [15]. The test level was α = 0.05 (bilateral).

## Results

### General data

The total number of patients who met the inclusion and exclusion criteria during the study time frame was 104 (RB:66 cases; RP: 38 cases), and the overall proportion of postoperative undernutrition was 28.85% (30/104, at risk of malnutrition:19 cases; malnutrition: 11 cases). The median age at the time of last nutritional assessment was 58.9 (50.9, 64.0) months (min: 41.1; max: 88.6 months). Among the cases, 42 (40.38%) were found to have CHD, with 17 cases (16.35%) receiving cardiovascular surgery. Of note, 27.88% of patients (29/104) underwent surgery for associated congenital anomalies (Table 1), of which cardiovascular surgery was the most common (58.62%). The detailed clinical characteristics of these patients were given in Table 2. Through analyzing the defecation function, we found that the proportions of poor, fair, good, and normal were 4.81%, 20.19%, 54.81%, and 20.19% in these patients, respectively. To avoid the influence of extreme values and facilitate statistical analysis, we grouped poor and fair defecation functions into one group (poor + fair).

**Table 1:** Details of the 29 patients received other surgeries

Associated organ systems	Cases (*n*/%)
Cardiovascular	10/34.5
Spinal/vertebral	7/24.1
Genitourinary	3/10.3
Cardiovascular + Genitourinary	2/6.9
Cardiovascular + Spinal/vertebral	3/10.3
Cardiovascular + Genitourinary + Spinal/vertebral	2/6.9
Genitourinary + Spinal/vertebral	2/6.9

**Table 2:** Relationship between patients’ general clinical characteristics and nutritional assessment

Factors	ALL patients (*N* = 104)	Normal (*N* = 74)	Undernutrition (*N* = 30)	*P* value
Type of ARM (n/%)ǂ				0.489
RB	66 (63.46)	49 (66.22)	17 (56.67)	
RP	38 (36.54)	25 (33.78)	13 (43.33)	
Birth weight (gram)^#^	2965 (2797.5, 3250)	2990(2800, 3250)	2900 (2790, 3287.5)	0.585
Gestational age (week)^#^	38.30 (37.27, 40.0)	38.30 (37.23, 40.0)	38.20 (37.32, 39.38)	0.345
Residence (*n*/%)ǂ				1.000
Urban	41 (39.42)	29 (39.19)	12 (40.0)	
Rural	63 (60.58)	45 (60.81)	18 (60.0)	
Feeding method (*n*/%)ǂ				< 0.001
Breast	44 (42.31)	38 (51.35)	6 (20.0)	
Mixed	33 (31.73)	25 (33.78)	8 (26.67)	
Artificial	27 (25.96)	11 (14.86)	16 (53.33)	
Relationship of caregiver (*n*/%)ǂ				< 0.001
Parents	82 (78.85)	66 (89.19)	16 (53.33)	
Others	22 (21.15)	8 (10.81)	14 (46.67)	
Educational level (*n*/%)ǂ				0.935
Primary and below	82 (78.85)	59 (79.73)	23 (76.67)	
Secondary and tertiary	22 (21.15)	15 (20.27)	7 (23.33)	
Age at the time of colostomy (hour, *n*/%)ǂ				0.333
< 24	53 (50.96)	41 (55.41)	12 (40.0)	
24 ~ < 48	38 (36.54)	24 (32.43)	14 (46.67)	
≥ 48	13 (12.50)	9 (12.16)	4 (13.33)	
Age at the time of PSARP (month)	5.80 (5.18, 6.80)	5.70 (5.20, 6.77)	6.10 (5.18, 7.55)	0.264
Nutritional status at PSARP (*n*/%)ǂ				0.130
Normal	75 (72.12)	57 (77.03)	18 (60.0)	
Undernutrition	29 (27.88)	17 (22.97)	12 (40.0)	
Age at the time of colostomy closure (month)^*^	9.20 ± 1.35	9.14 ± 1.36	9.35 ± 1.33	0.484
Nutritional status at colostomy closure (*n*/%)ǂ				0.119
Normal	69 (66.35)	53 (71.62)	16 (53.33)	
Undernutrition	35 (33.65)	21 (28.38)	14 (46.67)	
MCD (*n*/%)ǂ				1.000
No	43 (41.35)	31 (41.89)	12 (40.0)	
Yes	61 (58.65)	43 (58.11)	18 (60.0)	
CHD (*n*/%)ǂ				0.013
No	62 (59.62)	49 (66.22)	13 (43.33)	
Minor	25 (24.04)	18 (24.32)	7 (23.33)	
Major	17 (16.35)	7 (9.46)	10 (33.33)	
Other surgery *(n/%)ǂ*				0.013
Yes	29 (27.88)	15 (20.27)	14 (46.67)	
No	75 (72.12)	59 (79.73)	16 (53.33)	
Defecation function (*n*/%)ǂ				0.017
Poor + fair	26 (25.0)	13 (17.57)	13 (43.33)	
Good	57 (54.81)	46 (62.16)	11 (36.67)	
Normal	21 (20.19)	15 (20.27)	6 (20.00)	
Age at the time of last nutritional assessment (month)^#^	58.9 (50.9, 64.0)	59.4 (50.9, 63.8)	55.7 (52.3, 64.1)	0.931

*RB* rectobulbar, *RP* rectoprostatic, *MCD* multiple congenital disorders, *CHD* congenital heart defect, *PSARP* posterior sagittal anorectoplasty

ǂData was expressed by n (%) and used Chi-square test

^#^Values were presented as medians (IQR) and used Mann-Whitney U test

^*^Values were presented as mean ± standard deviation and used Student’s t test

### Correlation heatmap of the predictive variables

**Fig. 2:**
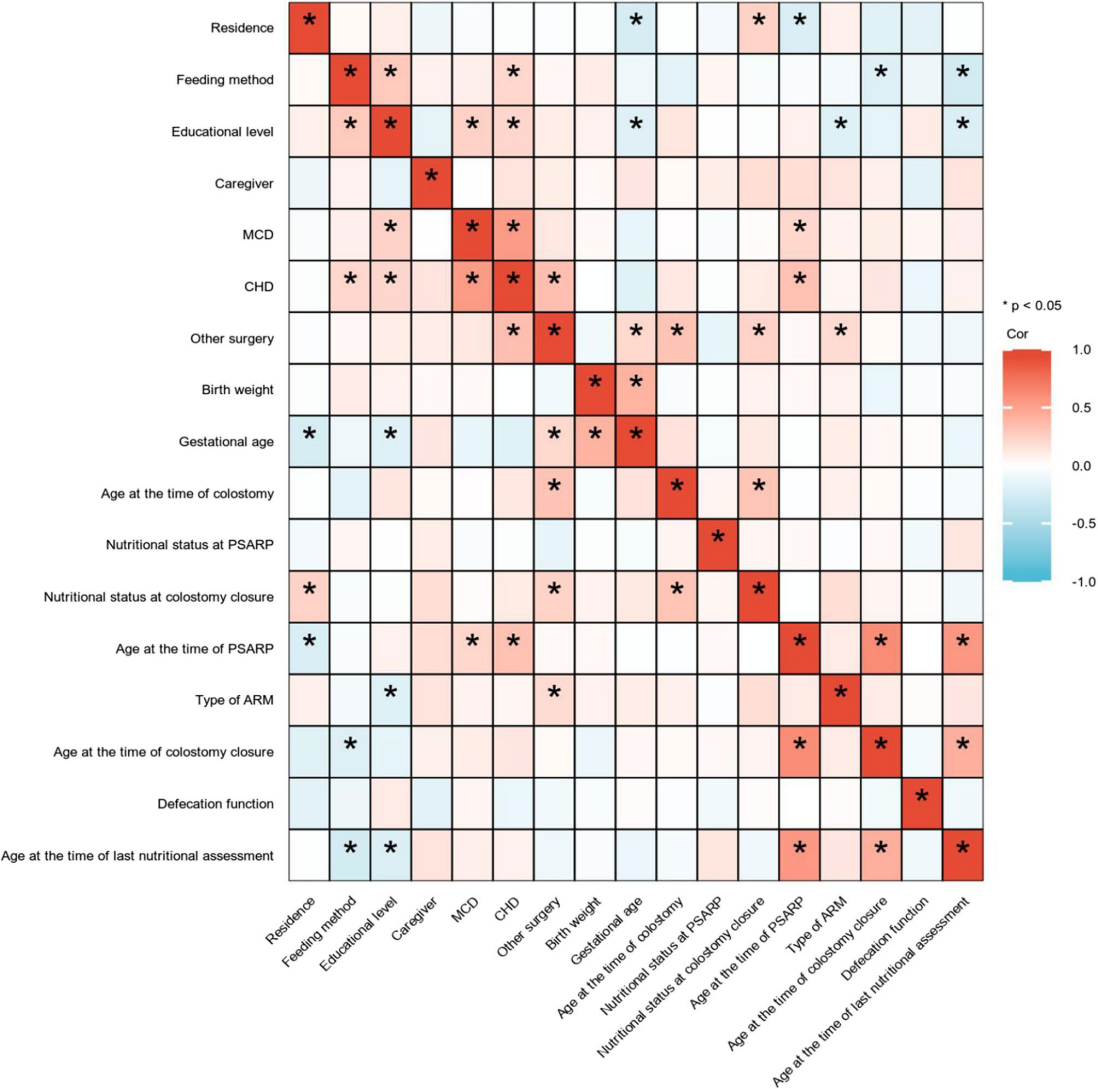
Correlation matrix of variables in the study population. *, *P* < 0.05

A correlation matrix of this cohort was shown in Fig. 2 to illustrate the relationships between various predictor variables, and it predominantly showed weak interactions, as indicated by the light-colored cells. However, the deep blue and red areas represented strong negative and positive correlations, respectively. For example, there was a significant positive correlation between the incidences of CHD and MCD, and a negative correlation between gestational age and residence. These patterns highlight how the study variables were interconnected and influence each other, reinforcing the need to factor in these relationships during our analysis.

### Screening for predictive factors of undernutrition

**Fig. 3:**
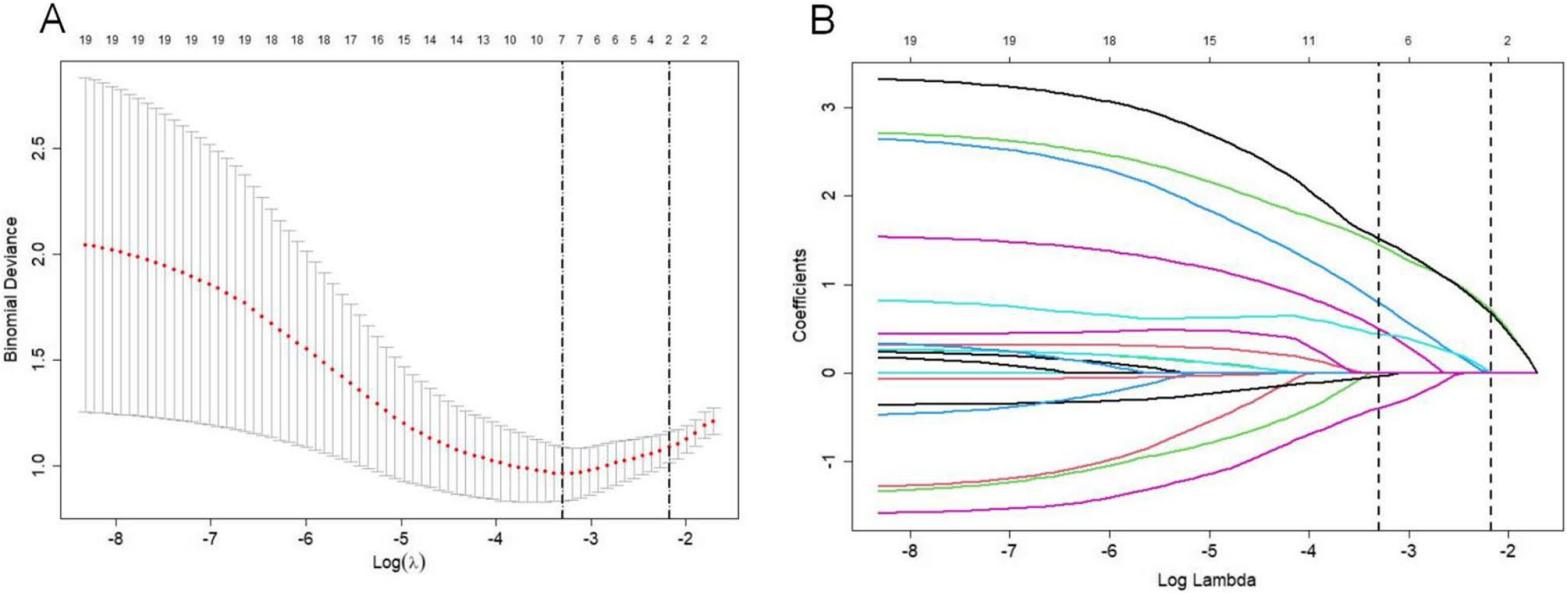
Screening of variables based on LASSO regression. (**A**) LASSO coefficients produced by the regression analysis. Coefficient profiles of 17 features plotted against log(λ). As the penalty increases, less informative variables shrink toward zero. (**B**) LASSO coefficient profiles of the 7 variables. Ten-fold cross-validation for tuning parameter selection. The left dashed line indicates the value of λ with minimum mean squared error; the right dashed line represents the largest λ within 1 standard error of the minimum. LASSO, least absolute shrinkage and selection operator

Considering the design characteristics of small sample size and multiple variables in this study, variable selection was performed using the LASSO regression model. Ten-fold cross-validation was conducted to identify the optimal penalty parameter (lambda. min) for the model and detected seven variables with nonzero coefficients: surgery for other systemic malformation, nutritional status at anorectoplasty, gestational age, defecation function, relationship of caregiver, CHD, and feeding method (Fig. 3A and B). Then, multivariate logistic regression model was constructed with these significant indicators and revealed four significant predictive factors for the development of Nomogram model: relationship of caregiver (95% CI: 2.806–52.99, OR: 11.20), nutritional status at anorectoplasty (95% CI: 1.087–17.06, OR: 4.101), surgery for other systemic malformations (95% CI: 1.902–45.40, OR: 8.378), and feeding method (reference: breast; artificial, OR: 95% CI: 4.927–194.2, OR: 25.32) (Table 3). These four factors were statistically significant (*P* < 0.05), validating their importance in predicting postoperative undernutrition. Diagnosis of collinearity for the above four variables was performed, and the variance inflation factors (VIF) were 1.027, 1.038, 1.038, and 1.009 (all VIFs < 5), respectively, indicating that there was no multiple collinearity relationship existed.

**Table 3:** Logistic regression analysis for potential influencing factors

Factors	β	SE	OR (95% CI)	*P*
Other surgery (Yes)	2.126	0.796	8.378 (1.902–45.40)	0.008
Nutritional status at PSARP (Undernutrition)	1.411	0.692	4.101 (1.087–17.06)	0.041
Gestational age	−0.224	0.167	0.799 (0.565–1.107)	0.180
Feeding methodǂ				
Mixed	1.119	0.775	3.061 (0.692–15.23)	0.149
Artificial	3.232	0.920	25.32 (4.927–194.2)	< 0.001
Relationship of caregiver (Others)	2.416	0.739	11.20 (2.806–52.99)	0.001
CHD#				
Minor	−0.775	0.820	0.460 (0.081–2.134)	0.345
Major	0.352	0.853	1.422 (0.260–7.764)	0.680
Defecation function*				
Good	−1.096	0.720	0.334 (0.076–1.346)	0.128
Normal	−0.175	0.930	0.839 (0.127–5.235)	0.851

*CHD* congenital heart defect, *β* regression coefficient, *SE* standard error, *OR* odds ratio, *95% CI* 95% confidence interval, *PSARP* posterior sagittal anorectoplasty

ǂBreast feeding as dummy variable

#No CHD as dummy variable

*Poor + fair defecation function as dummy variable

### Prediction model development

**Fig. 4:**
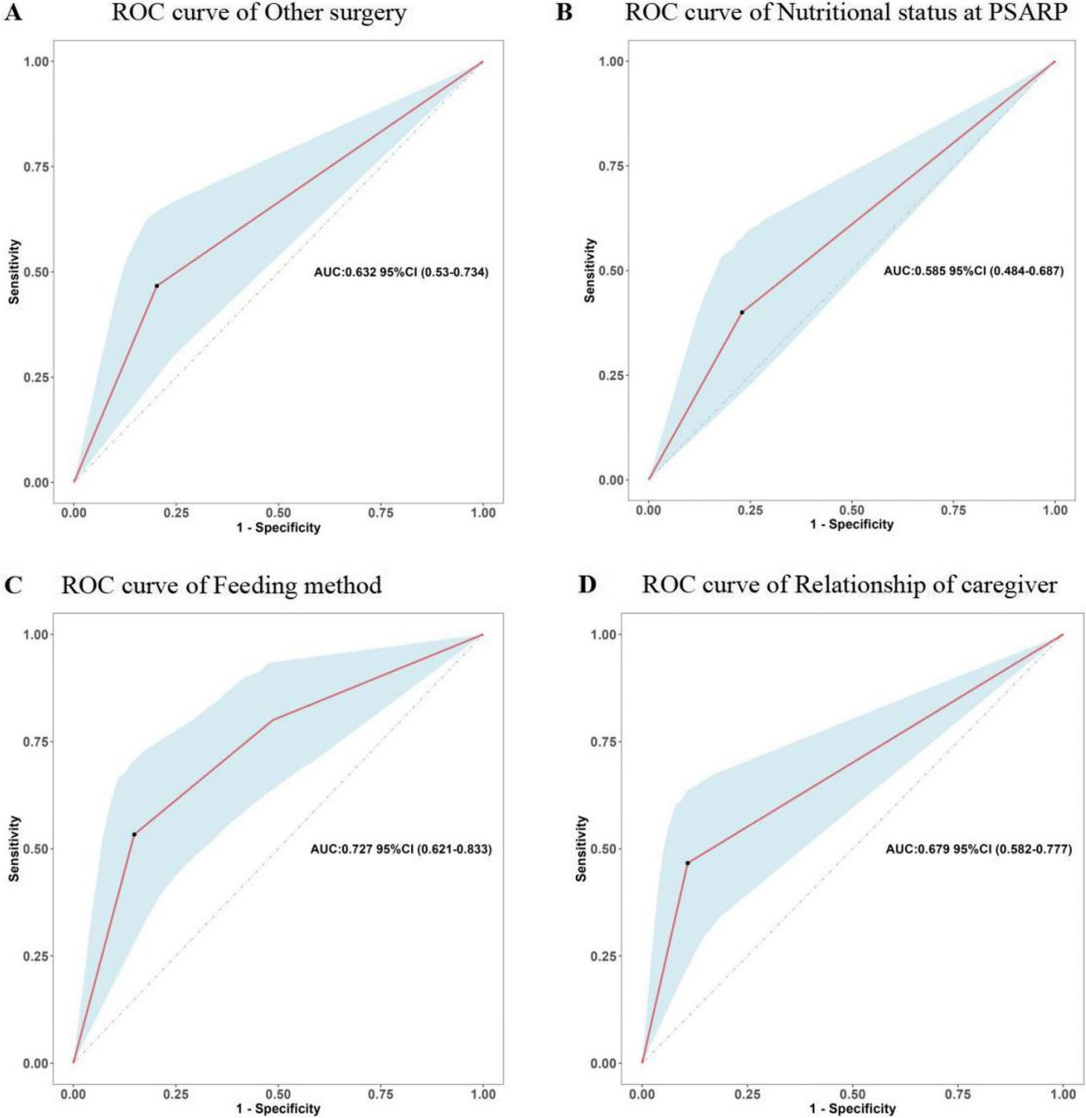
Predictive assessment of influencing factors for postoperative undernutrition with ROC curve analysis. (**A**) AUC for the other surgery (AUC = 0.63, 95% CI: 0.530–0.734). (**B**) AUC for the nutritional status at PSARP (AUC = 0.585, 95% CI: 0.484–0.687). (**C**) AUC for the feeding method (AUC = 0.727, 95% CI: 0.621–0.833). (**D**) AUC for the relationship of caregiver (AUC = 0.679, 95% CI: 0.582–0.777) ROC, receiver operating characteristics; AUC, area under the curve; PSARP, posterior sagittal anorectoplasty

Based on the Lasso-Logistic regression analysis results, we explored the predictive value of these factors for postoperative undernutrition. ROC curve analysis of relationship of caregiver, nutritional status at anorectoplasty, surgery for other systemic malformations, and feeding method resulted in AUCs of 0.679 (95% CI: 0.582–0.777), 0.585 (95% CI: 0.484–0.687), 0.632 (95% CI: 0.530–0.734), and 0.727 (95% CI: 0.621–0.833), respectively (Fig. 4). The specific predictive values of each factor were shown in Table 4. The feeding method showed a clearly better predictive performance for identifying the patients who were malnourished later, with 53.3% sensitivity, 85.1% specificity, 59.3% positive predictive value, and 81.8% negative predictive value.

**Table 4:** Predictive values of variables screened for postoperative undernutrition

Variable	AUC	SE	95% CI	Sensitivity (%)	Specificity (%)	PPV(%)	NPV(%)
Relationship of caregiver (Others)	0.679	0.050	0.582–0.777	46.7	89.2	63.6	80.5
Nutritional status at PSARP (Undernutrition)	0.585	0.052	0.484–0.687	40.0	77.0	62.5	80.3
Other surgery (Yes)	0.632	0.052	0.530–0.734	46.7	79.7	41.4	76.0
Feeding method (Artificial)	0.727	0.054	0.621–0.833	53.3	85.1	59.3	81.8

*AUC* area under the curve, *SE* standard error, *95% CI* 95% confidence interval, *PPV* positive predictive value, *NPV* negative predictive value, *PSARP* posterior sagittal anorectoplasty

**Fig. 5:**
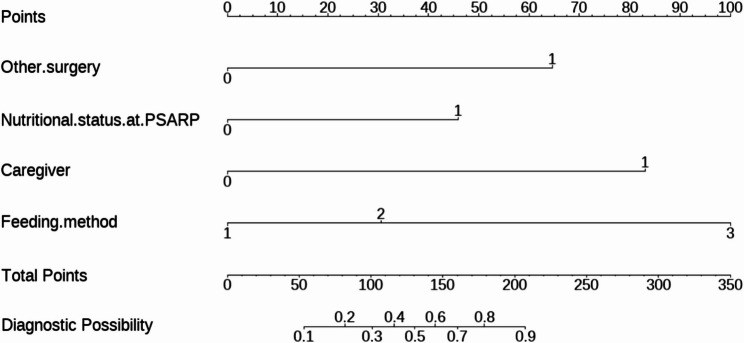
Nomogram model for predicting postoperative undernutrition. The assignment of each variable: caregiver: Others = 1, Parents = 0; undernutrition at PSARP: Yes = 1, No = 0; other surgery: Yes = 1, No = 0; feeding method: breast = 1, mixed = 2, artificial = 3. PSARP, posterior sagittal anorectoplasty

**Fig. 6:**
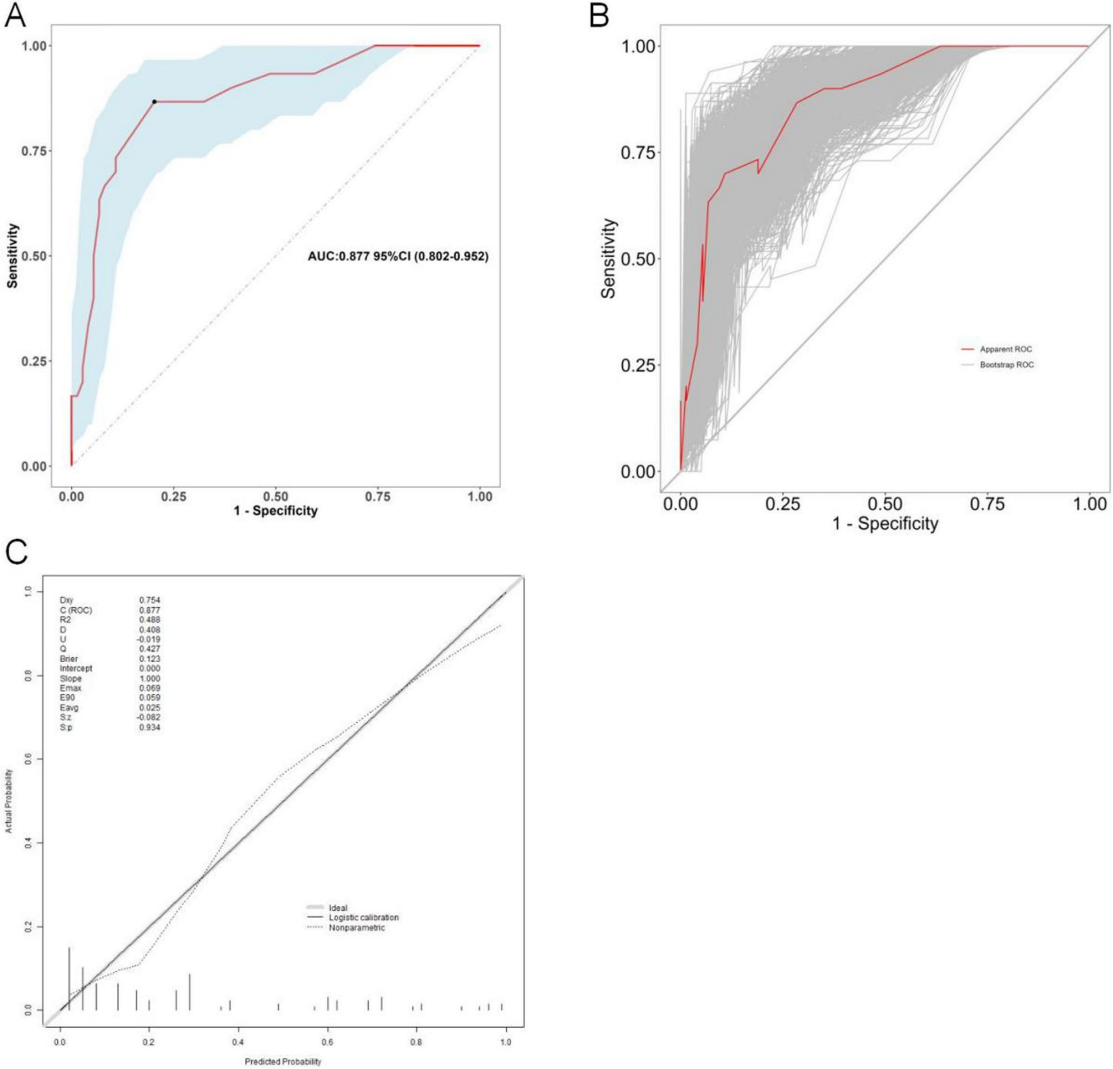
Prediction performance of Nomogram model. (**A**) AUC for the nomogram graph in the study cohort (AUC = 0.877, 95% CI: 0.802–0.952). (**B**) AUC for the nomogram graph in bootstrap resamples (AUC = 0.906, *n* = 1000, 95% CI: 0.843–0.969). (**C**) The calibration curves of the predictive model. ROC, receiver operating characteristic; AUC, area under the curve

From the above analysis, we found that the predictive value (sensitivity and specificity) of a single factor was not ideal, thus we established the Nomogram model (Fig. 5). The AUC for the predictive Nomogram model was 0.877 (95% CI: 0.802–0.952, Fig. 6A). To verify the accuracy of this model, a corrected AUC was calculated through 1,000 bootstrap resamples, with a desirable value of 0.906 (95% CI: 0.843–0.969, Fig. 6B). The results of H-L goodness-of-fit test showed that there was no statistically significant difference between the predicted and the actual incidence of malnutrition (χ^2^ = 7.554, *P* = 0.0.273). Meanwhile, the calibration curves indicated that the forecast was in good agreement with the actual situation (Fig. 6C). For example, a patient with cardiovascular surgery, undernutrition at PSARP, non-parental caregiver, and breast feeding, the corresponding score of each predictor was 64.5 points, 46 points, and 83 points respectively. The total score was 193.5 points, indicating that the risk of postoperative undernutrition was over 85% in this patient (Supplemental Figure S1).

To further stratify the risk of postoperative undernutrition, we applied the optimal cut-off function and the Youden index to calculate the binary risk stratification threshold of the prediction model in this cohort. We assessed two different thresholds for Diagnostic Probability, 0.27 and 0.5, which we named best and optimal cut-off value, respectively. We then used the intersection of the two thresholds to create three risk degree: low-risk, intermediate-risk, and high-risk groups. These corresponded to predicted probabilities of less than or equal to 0.27, greater than 0.27 and less than or equal to 0.5, and greater than 0.5, respectively.

**Fig. 7:**
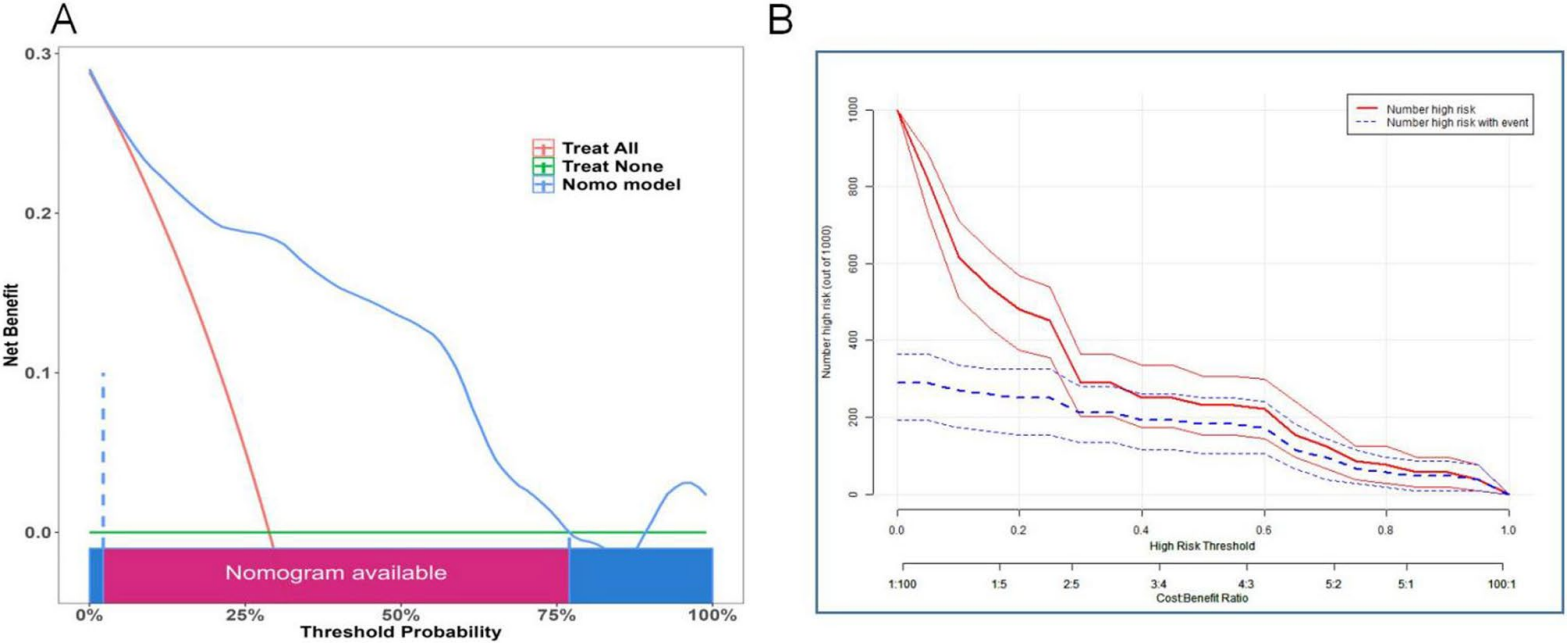
Decision curve analysis (DCA) and clinical impact curve (CIC) for predictive model. (**A**) The DCA of this cohort. In the DCA plots, Green-solid line: the patient does not apply the Nomogram and the net benefit is zero; Red-solid line: all patients are treated by the Nomogram. The area enclosed by the three lines presents the clinical utility of the Nomogram. (**B**) The CIC of the study population. In the CIC plots, the threshold probability exceeded 0.27, the predicted number of high-risk patients closely matched the actual number, suggesting that the model has good clinical utility in identifying high-risk individuals. DCA, decision curve analysis; CIC, clinical impact curve

Furthermore, The analysis of decision curve showed that when the probability threshold of Nomogram model predicting undernutrition was >0.03, application of the model would add net benefit compared to either the treat-all strategy or the treat-none strategy, as shown in Fig. 7A. The CIC was plotted to evaluate clinical significance (Fig. 7B). When the threshold probability exceeded 0.27, the two curves became very close, indicating that the number of high-risk patients predicted by the model was very close to the actual number of high-risk patients. The results of binary risk stratification threshold and CIC analysis were consistent, indicating that when the diagnostic probability was higher than 0.27, taking corresponding intervention protocols may be benefits for patients. In summary, this model exhibited excellent prediction performance.

**Fig. 8:**
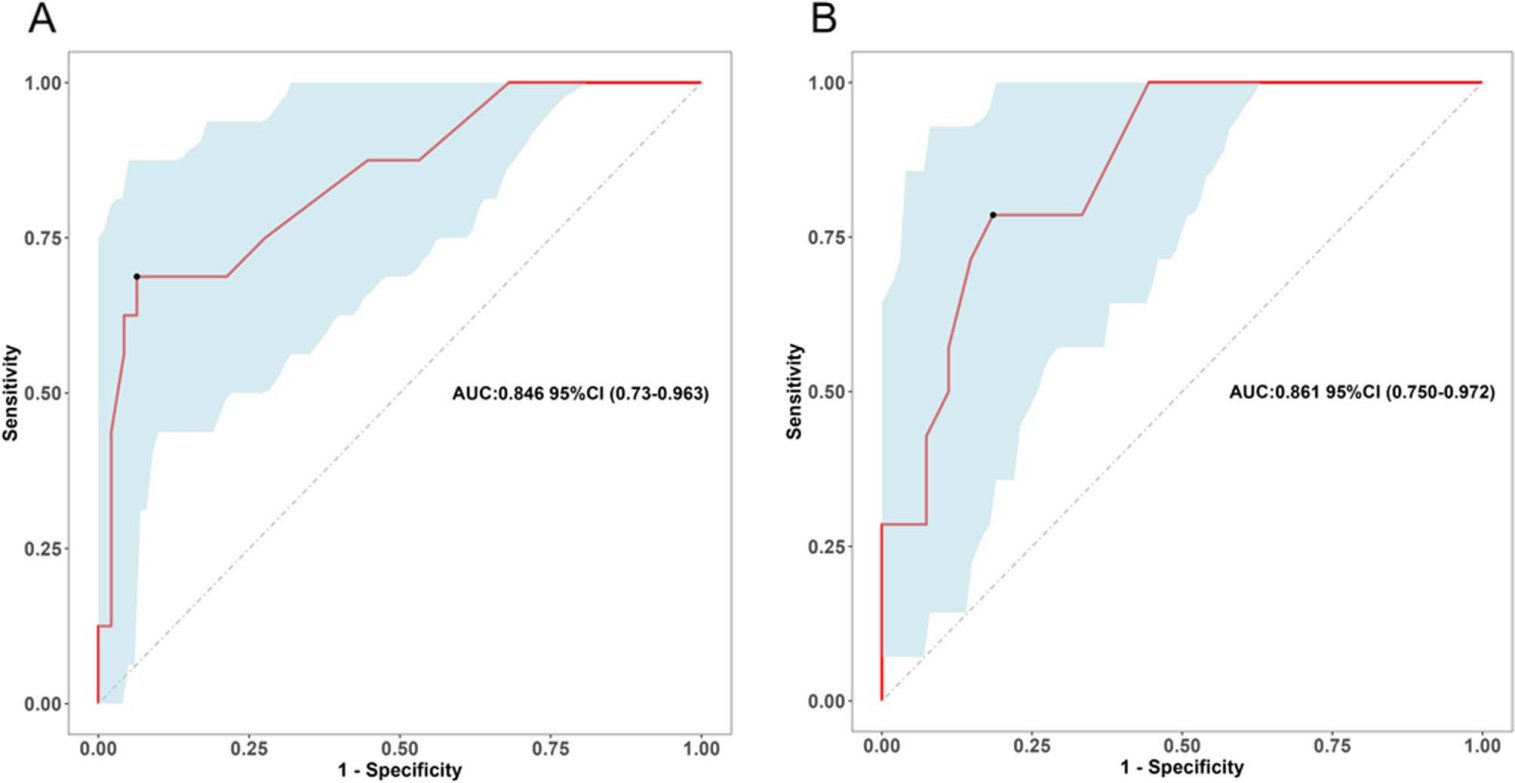
Predictive assessment of Nomogram model for different time frame with ROC curve analysis. (**A**) AUC for patients from June 2015 to December 2017 (AUC = 0.846, 95% CI: 0.730–0.963). (**B**) AUC for patients from January 2018 to November 2020 (AUC = 0.861, 95% CI: 0.750–0.972). AUC, area under the curve

Due to the long time span of this study, we have divided these patients into two groups (Group 1: June 2015 to December 2017, 63 cases, 16 for undernutrition; Group 2: January 2018 to November 2020, 41 cases, 14 for undernutrition) according to the time period and performed temporal validation. The AUCs of this model in Group 1 and 2 were 0.846 (95% CI: 0.730–0.963) and 0.861 (95% CI: 0.750–0.972), respectively (Fig. 8), indicating that the model has good stability in different time periods.

## Discussion

Male complex ARM is a challenge for pediatric surgeons (accounting for about 90% of the total complex ARM), which mainly divides into the following three forms: imperforate anus with RB, RP, and rectobladderneck fistulas [16]. The purposes of surgery include anal anatomical reconstruction, defecation function recovery, mental health, and normal development. During the study period, five cases with rectobladderneck fistula received laparoscopically assisted anorectoplasty in our department, which was not included in this analysis due to the small sample size and inconsistent surgical strategies. Postoperative defecation function and quality of life have always been the focus of our team’s regular follow-up of patients, but during this process, we found that the nutritional problems were not optimistic. These malnourished patients experience ongoing health challenges, unable to lead a normal life, placing a heavy financial and psychological burden on themselves and their families [17]. Currently, little relevant studies have reported the problem of postoperative undernutrition in patients with complex ARM. Therefore, we conducted this study to investigate the current status of postoperative undernutrition and established prediction model.

Nutritional problems after anorectoplasty in patients with ARM were usually overlooked. This is the first study to identify that the proportion of postoperative undernutrition was 28.85% in male complex ARM. The correlation heatmap revealed notable deep blue and red spots, representing strong positive or negative correlations between specific variables, e.g. a significant positive correlation between CHD and MCD and a negative correlation between gestational age and residence. These correlations highlighted the interdependence between variables, emphasizing the importance of considering these relationships in our analysis. Thus, we used LASSO-Logistic regression analysis to systematically identified the most relevant predictors of postoperative undernutrition. This method ensures model parsimony and robustness, effectively avoiding over-fitting while maintaining high predictive accuracy [18, 19]. The logistic regression analysis confirmed a more refined set of predictors [20, 21], confirming their independent and significant contributions to postoperative undernutrition. In this way, the result showed that relationship of caregiver, nutritional status at anorectoplasty, surgery for other systemic malformations, and feeding method were independently associated with postoperative undernutrition in patients with complex ARM. Furthermore, we established a Nomogram model based on these four factors, aiming to predict postoperative undernutrition and provide a reference for prevention and treatment in clinical practice. The model showed ideal predictive accuracy and might be used to aid the differentiation of not malnourished and malnourished patients for individualized intervention.

The results established a strong association between the relationship of caregivers and postoperative nutritional status, that is, patients with non-parental caregivers were more likely to develop postoperative undernutrition, in accordance with our expectations. However, apart from relationship of caregivers, residence and education level have also been reported to affect children’s nutritional status in large sample population studies [22–24], which was inconsistent with the results of this study. During the follow-up, we found that patients with non-parental caregivers usually had insufficient family economic conditions, and these non-parental caregivers, mainly grandparents, often failed to provide patients with a balanced nutritional requirement due to insufficient understanding of the disease and the influence of traditional concepts [25, 26]. Conversely, patients with parental caregivers may receive more attentive care and individualized feeding [27]. In clinical practice, we strongly recommend that patients be cared for by their parents, as we empirically believe that parents are able to be more attentive to individualized feeding and improve postoperative nutritional status.

The study found that patient who underwent surgery for other systemic malformations was more likely to suffer from postoperative undernutrition. In this study, 29 patients underwent surgery for other systemic malformations, among which cardiovascular surgery was the most common (58.6%). The severity of CHD was found to be related to postoperative nutritional status in LASSO analysis, which was consistent with the results reported in other studies [28–30]. Due to limited heart function and unstable blood flow available for the gastrointestinal tract, patients with CHDs often experience reduced appetite, increased energy consumption, and impaired nutrient absorption, resulting in weight loss and undernutrition [31]. Generally speaking, the more severe CHD is, the greater its adverse impact on nutritional status [32]. It should be noted that major CHD was usually treated with surgery, so this interaction caused that only “surgery for other systemic malformation” was found to be an independent factor for postoperative undernutrition in Logistic regression analysis. After multiple surgeries, the body requires additional energy and nutrients for repair and recovery, and this process can lead to an imbalance of nutrients without individualized nutritional management [33]. Therefore, pediatric surgeons and dietitians should attach importance to the nutritional management of patients who have undergone multiple surgeries, including the nutritional assessment and support during the perioperative period of each surgery.

It is well established that preoperative undernutrition is a major risk factor for increased postoperative morbidity and mortality, and an improved preoperative nutritional status leads to favorable surgical outcomes [34]. In this study, all patients underwent three-staged repair, among which the most important and complex one was anorectoplasty. Of course, it suggests that the preoperative nutritional status at this stage is the most important for clinical outcomes, compared with the nutritional status at other surgical stages. This surgical shock could cause stress and metabolic changes in the body, e.g. catabolism of glycogen, fat and protein, further aggravate the postoperative nutrition problem of those patients [35]. Due to the fact that patients with complex ARM received colostomy after birth, resulting in insufficient nutrient intake and/or increased energy consumption, there is a relatively high risk of undernutrition before the second-stage surgery. Based upon these findings, our research conducted a more detailed observation of the preoperative- and postoperative- nutritional status. We found that nutritional status at anorectoplasty is an independent predictor for postoperative undernutrition. Therefore, optimal the nutritional status before anorectoplasty is of great significance for improving the surgical outcome and prognosis [36].

Early life nutrition is fundamental to children’s growth and development [37]. Over the past few decades, there has been increasing evidences showed that breast feeding can not only meet the nutritional needs of infant growth and development, but also promote infant organ development and functional maturity [38]. Breast feeding, compared to other feeding methods, especially formula feeding, has been shown to ensure the best health and developmental outcomes for infants [39]. Furthermore, substantive evidence shows early nutrition (breast feeding for the first six months of life) influences development of the microbiome and immune system, affecting lifelong health [40]. This study found that patients who received artificial feeding were more likely to suffer from postoperative undernutrition than those who were breastfed. In this cohort, the breast feeding rate in patients with ARM was 42.31% (44/104), similar with the overall rate of breast feeding in our country [41]. This study has enriched the benefits of breast feeding in patients with ARM, and its improvement for clinical outcomes is noteworthy. Hence, the feeding methods for patients with complex ARM require more attention, and breast feeding should be recommended for these patients to improve the postoperative nutritional status.

Finally, we developed the Nomogram model based on the above four independent factors to better assess the possibility of postoperative undernutrition, so that we could fully communicate postoperative complications with families and provide limited medical resources to high-risk populations. In addition, this was based on the idea that it might be of value to provide a reference for early prevention and treatment. ROC curve analysis showed that the AUC of Nomogram model for predicting postoperative undernutrition was 0.877 (95% CI: 0.802–0.952). The AUC was calculated through 1,000 bootstrap resamples, with a desirable value of 0.906. And the analysis of H-L goodness-of-fit test and calibration curve showed that this model had desirable prediction efficiency, which is helpful for clinicians to identify high-risk patients early. It is recommended that the new prediction model needs to be verified by validation samples of the center or other centers to truly reflect the prediction performance of the model [42]. Inadequately, this model is only validated in the same cohort from which the Nomogram model was established, it has not been validate the prediction model in another patient cohort with large sample size.

## Limitation

However, this study has several limitations. First, this analysis was retrospective and conducted in one hospital with a relatively small cohort, the diversity of patients in terms of geographic, ethnic, and clinical practice characteristics was restricted. Further research with a larger prospective cohort study is needed to validate or optimize the accuracy of the model. Second, several variables (i.e. short-term postoperative complications) were not included in the analysis due to the small event counts. Furthermore, data collection (i.e.wealth index) may be not comprehensive, and other influencing factors with statistical differences may not been shown in this analysis. Therefore, these results should be interpreted cautiously, and prospective multi-center study with a larger cohort are needed to assess the potential interaction effects for maintaining model simplicity and preventing overfitting. Third, considering that most of the patients included in this study originated from poor mountainous areas in southwestern China, with a relatively high prevalence of postoperative undernutrition (28.85%), which is substantially higher than might be expected in more affluent healthcare settings, raising questions about the applicability of the Nomogram to other populations. Finally, due to the limited sample size, we only established the prediction model, and external validation is still required to further evaluate the performance of the Nomogram model.

## Conclusions

To our knowledge, this is the first study to identified that relationship of caregiver, nutritional status at anorectoplasty, surgery for other systemic malformations, and feeding method were independently associated with postoperative undernutrition, and the Nomogram model based on these determinants showed desirable discrimination to assess the individualized prediction. This finding can be used by pediatric surgeons to early identify patients at high risk of postoperative undernutrition, to take early preventive and curative strategies, and to provide reference for guiding parental counseling. However, further studies including external validation, are required to assess the predictive performance of the model in another cohort.

## Supplementary Information

Supplementary Material 1.

## Data Availability

The datasets generated and analyzed during the current study are not publicly available due to the ongoing analysis in other directions but are available from the corresponding author (Rui Jiang) on reasonable request.

## Abbreviations

ARM: Anorectal malformation
RB: Rectobulbar fistula
RP: Rectoprostatic fistula
CHD: Congenital heart defect
MCD: Multiple congenital disorders
HAZ: Height-for-age
WAZ: Weight-for-age
SD: Standard deviation
LASSO: Least absolute shrinkage and selection operator
ROC: Receiver operating characteristic curve
DCA: Decision curve analysis
CIC: Clinical impact curve
CI: Confidence interval
ACU: Area under the curve
OR: odds ratio
