# Supplementary material for: Development of nomogram model based on LASSO-Logistic regression for predicting postoperative undernutrition to complex anorectal malformation: a pilot exploratory study
Source: BMC Gastroenterol. 2025 Aug 16;25:588. doi: 10.1186/s12876-025-04202-5 (PMC12357407; doi:10.1186/s12876-025-04202-5)
Supplement: Supplementary file 1 — Supplementary Material 1. [file 12876_2025_4202_MOESM1_ESM.docx]

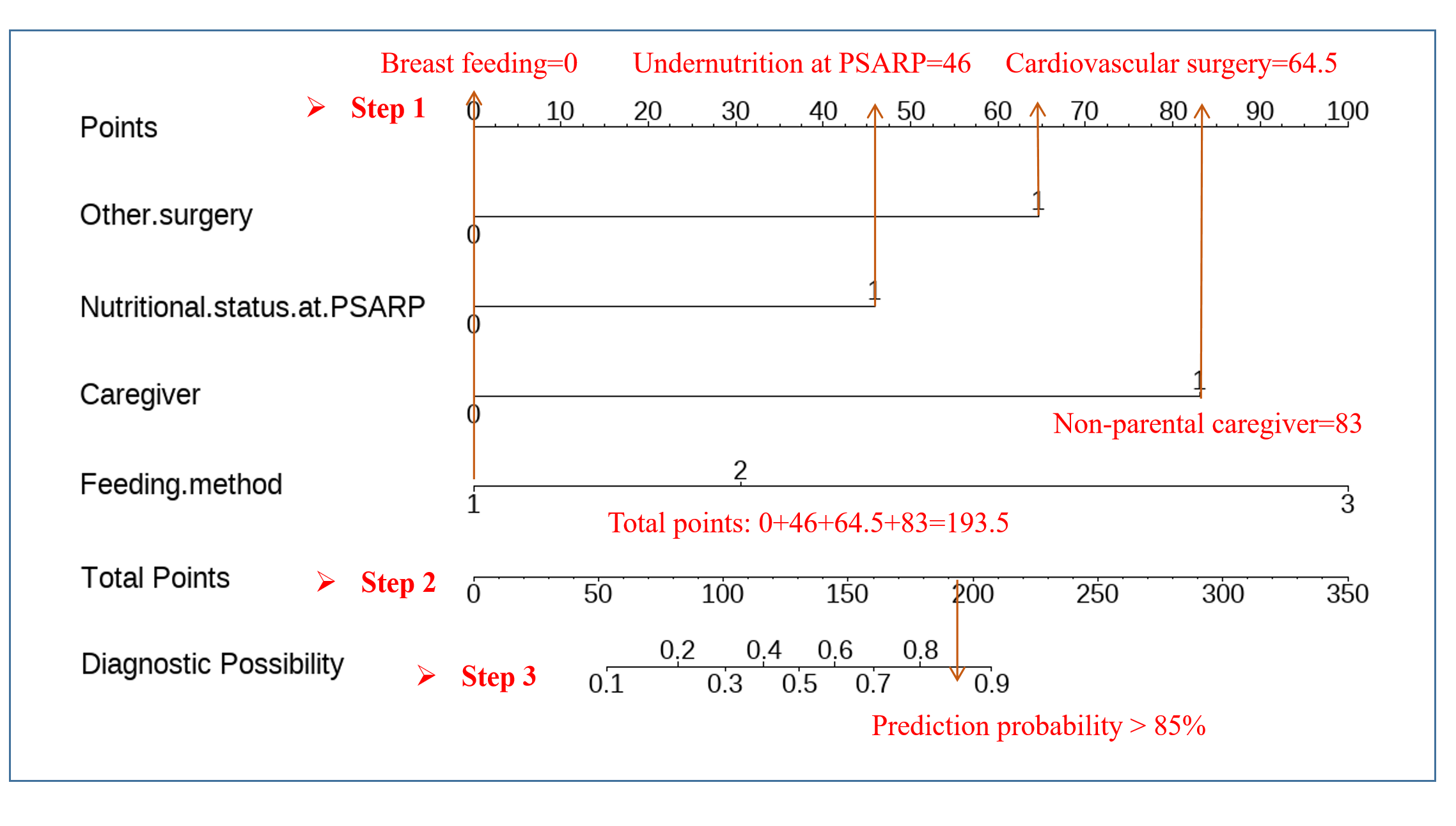


**Supplemental Figure S1.** A detailed example for nomogram model. For example, a patient with cardiovascular surgery, undernutrition at PSARP, non-parental caregiver, and breast feeding, the corresponding score of each predictor was 64.5 points, 46 points, and 83 points respectively. The total score was 193.5 points, indicating that the risk of postoperative undernutrition was over 85% in this patient. PSARP, posterior sagittal anorectoplasty.
